# The grass is not always greener: a multi-institutional pilot study of marijuana use and acute pain management following traumatic injury

**DOI:** 10.1186/s13037-018-0163-3

**Published:** 2018-06-19

**Authors:** Kristin Salottolo, Laura Peck, Allen Tanner II, Matthew M. Carrick, Robert Madayag, Emmett McGuire, David Bar-Or

**Affiliations:** 1 Trauma Research Department, Swedish Medical Center, 501 E. Hampden Ave, Englewood, CO 80113 USA; 2 Trauma Research Department, St. Anthony Hospital, 11600 W. 2nd Place, Lakewood, CO 80228 USA; 3 Trauma Research Department, Medical City Plano, 3901 West 15th Street, Plano, TX 75075 USA; 4 Trauma Research Department, Penrose Hospital, 2222 N Nevada Drive, Colorado Springs, CO 80907 USA; 5 Trauma Services Department, Swedish Medical Center, 499 E. Hampden Ave, Englewood, CO 80113 USA; 6 Trauma Services Department, Penrose Hospital, 2222 N Nevada Drive, Colorado Springs, CO 80907 USA; 7 Trauma Services Department, Medical City Plano, 3901 W 15th St, Plano, TX 75075 USA; 8 Trauma Services Department, St. Anthony Hospital, 11600 West 2nd Place, Lakewood, CO 80228 USA

**Keywords:** Marijuana, Acute pain management, Vehicular trauma, Substance abuse

## Abstract

**Background:**

Widespread legislative efforts to legalize marijuana have increased the prevalence of marijuana use and abuse. The effects of marijuana on pain tolerance and analgesic pain management in the acute pain setting have not been reported. Although marijuana has been shown to have antinociceptive effects and is approved for medical use to treat chronic pain, anecdotal evidence suggests marijuana users admitted with traumatic injuries experience poorer pain control than patients who do not use marijuana. We hypothesized that marijuana users would report higher pain scores and require more opioid analgesia following traumatic injury.

**Methods:**

This retrospective pilot study included all patients involved in motor vehicle crashes, consecutively admitted to four trauma centers from 1/1/2016–4/30/2016. Marijuana status was examined as non-use and use, and was further categorized as chronic and episodic use. We performed a repeated measures mixed model to examine the association between marijuana use and a) average daily opioid consumption and b) average daily pain scores (scale 0–10). Opioid analgesics were converted to be equianalgesic to 1 mg IV hydromorphone.

**Results:**

Marijuana use was reported in 21% (54/261), of which 30% reported chronic use (16/54). Marijuana use was reported more frequently in Colorado hospitals (23–29%) compared to the hospital in Texas (6%). Drug use with other prescription/street drugs was reported in 9% of patients. Other drug use was a significant effect modifier and results were presented after stratification by drug use. After adjustment, marijuana users who did not use other drugs consumed significantly more opioids (7.6 mg vs. 5.6 mg, *p* <  0.001) and reported higher pain scores (4.9 vs. 4.2, *p* <  0.001) than non-marijuana users. Conversely, in patients who used other drugs, there were no differences in opioid consumption (5.6 mg vs. 6.1 mg, *p* = 0.70) or pain scores (5.3 vs. 6.0, *p* = 0.07) with marijuana use compared to non-use, after adjustment. Chronic marijuana use was associated with significantly higher opioid consumption compared to episodic marijuana use in concomitant drug users (11.3 mg vs. 4.4 mg, *p* = 0.008) but was similar in non-drug users (*p* = 0.41).

**Conclusion:**

These preliminary data suggest that marijuana use, especially chronic use, may affect pain response to injury by requiring greater use of opioid analgesia. These results were less pronounced in patients who used other drugs.

## Background

Substance abuse in the United States is on the rise, especially marijuana, ranking first in prevalence after alcohol [1]. Acute pain management among patients with substance abuse problems is challenging for numerous reasons, including drug cross-tolerance effects [2–4] and opioid-induced hyperalgesia and withdrawal [5, 6]. The effects of marijuana use and abuse on acute pain management have not been addressed in prior studies and are poorly understood.

Marijuana was first used in the United States for alleviation of pain and spasticity [7]. Marijuana has recently been legalized in 29 states and the District of Columbia for medical and recreational use, due to the asserted medicinal effects and the perception of being a safe illicit substance. Marijuana’s medicinal effects are largely antinociceptive, suggesting a role in treating chronic pain [8–11] and neuropathic pain [12, 13]. Other purported benefits include treatment of the signs and symptoms of multiple sclerosis, rheumatoid arthritis, glaucoma, epilepsy, inflammatory bowel disease, and emesis in HIV and cancer patients [14, 15]. However, there is only limited evidence from randomized controlled trials that marijuana and cannabinoids are effective for these latter proposed benefits [16].

It is necessary to understand how marijuana use affects pain management following traumatic injury for two reasons: first, the rapid proliferation of States legalizing marijuana for both medical and recreational use has increased its availability and use [17, 18]. Over 22 million people report using marijuana within the past month [1], of which 40.3% use it regularly (on 20 or more days). Second, patients with traumatic injuries have a higher prevalence of drug use than the general population, reported in 40–50% of trauma patients [19, 20], and the likelihood of a positive drug toxicology screen following trauma has increased over time [21]. Moreover, the risk of traffic accidents is increased more than two-fold in patients testing positive for marijuana [22], with an increase in fatal motor vehicle crashes (MVCs) since the legalization of medical marijuana in Colorado [23].

Although marijuana has been shown to have antinociceptive effects, anecdotal evidence at two of the included institutions suggests poorer pain control in marijuana users admitted with traumatic injuries. The objective of this study was to determine if there is an association between pre-injury marijuana use and pain response following traumatic injury.

## Methods

The aims of the multi-institutional pilot study were to determine if there were differences, by marijuana use, in total daily opioid analgesics consumed and average daily pain scores.

The study population was identified from the Trauma Registry (TraumaBase®; CDM, Evergreen, CO). Study inclusion criteria consisted of adults (Age ≥ 18) admitted with a MVC (motor vehicle passenger/driver, motorcycle, auto-pedestrian) between January 1, 2016 – April 30, 2016 to four trauma centers: three level I centers (two centers in the Denver, CO metropolitan area and one in Plano, TX), and one level II trauma center located in Colorado Springs, CO. Patients with a hospital length of stay (LOS) > 14 days were excluded. The study was approved from the Institutional Review Boards at each hospital.

The following were abstracted from the trauma registry in real time by dedicated trauma registrars for all trauma patients: demographics (age, gender), arrival blood alcohol concentration (BAC, ≥ 80 mg/dl was defined as intoxication), urine drug screen (UDS) performed (yes/no) as well as positive results on the standard multi-drug UDS panel (positive for any of the following: amphetamines, barbiturates, benzodiazepines, cocaine, methamphetamine, opiates, and marijuana (tetrahydrocannabinol [THC]), injury severity score (ISS), injury mechanism (motor vehicle, motorcycle, auto-pedestrian), admission Glasgow coma scale (GCS) score, and clinical outcomes (mortality, hospital LOS, intensive care unit (ICU) LOS).

The following substance use information was abstracted from the electronic medial record: current substance use obtained from patient history and drug and alcohol screening with the screening, brief intervention, and referral to treatment (SBIRT) screen, CAGE questionnaire, or Alcohol, Smoking and Substance Involvement Screening Test (ASSIST); frequency of marijuana use, usually recorded as times used per day / week / month; amount of marijuana used, recorded as amount of “joints”, vapor hits, “edibles”, etc.

Additional information abstracted from the electronic medical record included: pain scores using standardized pain numeric rating scale (NRS, 0–10 scale), recorded as date and time and score; analgesic medication use, including date and time, dose, and route of administration; analgesics received at discharge, including dose and route of administration. Pain medication orders are similar in the ICU and on the medical floor. Standard practice is for patients to rate their pain every time an analgesic is administered and when analgesic orders are adjusted.

### Analysis

All analyses were performed using SAS version 9.3 (Cary, NC). Marijuana use was defined as self-reported current use of marijuana or as a positive UDS result for marijuana (THC). Chronic marijuana use was defined as self-reported daily or almost daily use or more than one ounce of marijuana over the past month. All other marijuana users were considered episodic users. Drug use, other than marijuana, was defined as self-reported current use/abuse of street or prescription drugs or a positive UDS result for amphetamines, barbiturates, benzodiazepines, cocaine, methamphetamines, opiates, or PCP.

**Fig. 1:**
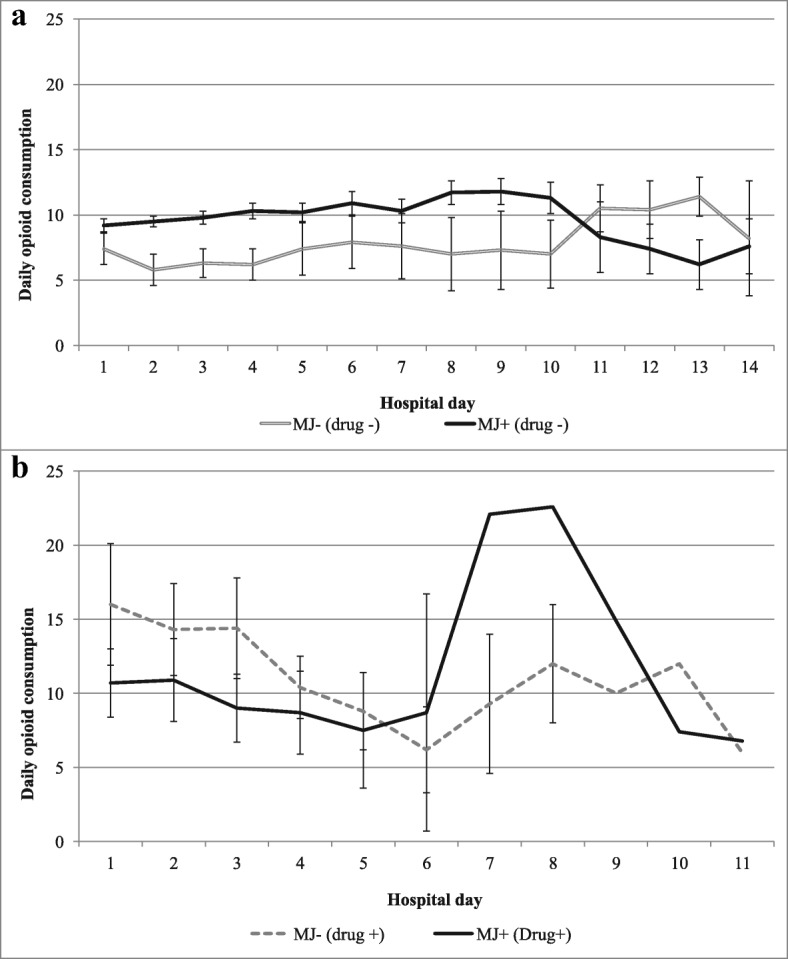
Average total daily opioid analgesics consumed, by marijuana (MJ) use in **a** non-drug users and **b** drug users (amphetamines, barbiturates, benzodiazepines, cocaine, opiates, PCP, or methamphetamine). Opioids were converted to be equianalgesic to 1 mg hydromorphone

**Fig. 2:**
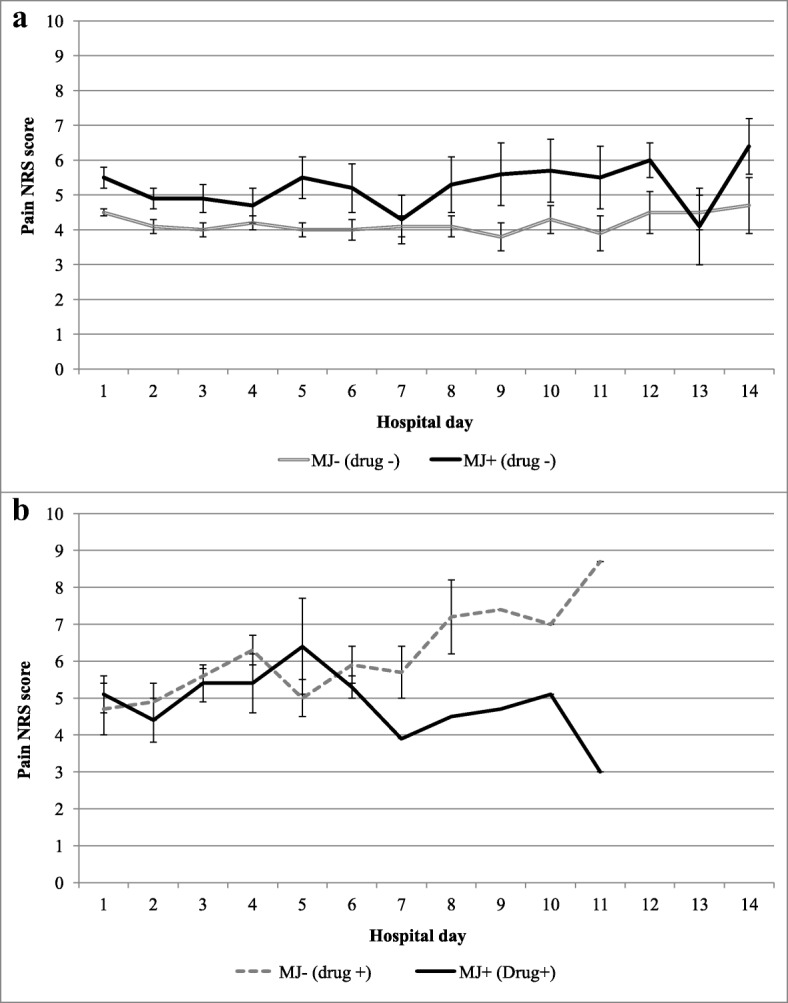
Average daily pain numeric rating scale (NRS) scores by marijuana (MJ) use in **a** non-drug users and **b** drug users (amphetamines, barbiturates, benzodiazepines, cocaine, methamphetamine, opiates). Patients are instructed that pain scores should be four or less, where scores of zero are not expected

Opioids are the mainstay analgesics for managing pain in the traumatic and critical care setting due to their proven efficacy in treating moderate to severe acute pain [24, 25]. Analgesics were classified as opioids and non-opioids, and all opioids were converted to be equianalgesic to 1 mg hydromorphone using an equianalgesic conversion chart. The amount of opioids consumed was summed for each 24-h period from admission through discharge; this total daily opioid count takes into account dose and frequency (Fig. 1). A square root transformation was used for opioid consumption to ensure a normal distribution in the final models; results are presented using back-transformed data. Pain scores were normally distributed; average daily pain NRS scores were calculated for each 24-h period from admission through discharge (Fig. 2).

Differences, by marijuana status, in total daily opioid analgesics consumed and average daily pain scores were examined with repeated measures mixed models. We tested for possible interactions for study covariates; drug use was a significant effect modifier, and all final models were seperately examined and reported for drug users (i.e. amphetamines, barbiturates, benzodiazepines, cocaine, methamphetamines, opiates, or PCP) and non-drug users. Final models are presented before adjustment and after adjustment for ISS, age, and specific cause of injury. All models were performed in marijuana users vs. non-using patients, and then chronic marijuana use vs. episodic use vs. non-users. Statistical significance was set at *p* <  0.05.

## Results

Marijuana use was reported in 21% (54/261) of patients, of which 30% reported chronic use (16/54). Marijuana use was reported more frequently in Colorado hospitals (23–29%) compared to the hospital in Texas (6%). The majority of patients did not specify the indication for use (51%); in those who did, 78% reported recreational use and 22% reported medical use. Compared to compared to non-users, marijuana userswere more likely to be younger (28 years vs. 46 years), and use drugs besides marijuana (26% vs. 4%), Table 1. There were no differences by marijuana status in gender, cause of vehicular trauma, GCS, ISS, alcohol intoxication, and no differences in any of the clinical outcomes (Table 1). There were also no differences in demographics, injury characteristics, and clinical outcomes between chronic and episodic marijuana users (Table 1).

**Table 1:** Characteristics and outcomes, by marijuana status

Characteristic (%, n)	Marijuana user *n* = 54	Non-user *n* = 207	*p* value	Chronic marijuana user *n* = 15	Episodic marijuana use *n* = 38	*p* value
Male sex	61% (33)	61% (126)	0.97	63% (10)	61% (23)	0.89
Age, years^a^	28 (23–39)	46 (26–65)	*< 0.001*	23 (22–43)	29 (23–38)	0.37
Cause of injury			0.08			0.43
Motor vehicle	80% (43)	80% (166)		69% (11)	84% (32)	
Motorcycle	4% (2)	11% (23)		6% (1)	3% (1)	
Pedestrian	17% (9)	9% (18)		25% (4)	13% (5)	
Normal Glasgow coma score 15	83% (45)	81% (168)	0.71	75% (12)	87% (33)	0.42
Injury severity score ISS^a^	9.5 (4–17)	9 (5–14)	0.74	7 (3.5–15.5)	10 (4–17)	0.37
Intoxicated (BAC ≥ 80 mg/dL)	26% (11)	25% (35)	0.98	31% (4)	23% (7)	0.71
Urine drug screen (UDS) performed	77% (41)	68% (135)	0.17	81% (13)	76% (28)	0.74
Positive UDS for drugs^b^	22% (12)	2% (5)	*< 0.001*	13% (2)	26% (10)	0.47
Drugs user (UDS/self-report)^b^	26% (14)	4% (9)	*< 0.001*	13% (2)	32% (12)	0.19
Clinical outcome						
Mortality	0% (0)	2% (5)	0.59	0% (0)	0% (0)	–
LOS, days^a^	2 (1–5)	3 (1–6)	0.57	3 (2–6)	2 (1–4)	0.35
ICU LOS, days^a^	1.5 (0–4)	2 (0–5)	0.81	1.5 (0–3)	1.5 (0–4.5)	0.89

*MJ* marijuana, *ICU* intensive care unit, *LOS* length of stay

^a^Data presented as median (IQR)

^b^Drugs: amphetamines, barbiturates, benzodiazepines, cocaine, methamphetamine, opiates

*P* < 0.05 in italic is clinically significant

In total 5863 analgesic doses were consumed over the hospitalization; the majority (86%) of the analgesics were opioids. The most commonly administered opioids were hydromorphone (dilaudid), accounting for 27% of all opioids consumed, oxycodone (26%), hydrocodone (11%) fentanyl (10%), morphine (10%), and tramadol (2%); codeine, methadone, meperedine, and nalbuphine were prescribed < 1% of the time. Throughout the hospitalization there were 7345 pain scores recorded, or an average of 7.7 pain assessments/patient/day.

### Results, non-drug users

Overall, 91% of patients did not test positive for drugs (i.e. amphetamines, barbiturates, benzodiazepines, cocaine, methamphetamine, or opiates). In this majority, marijuana users received significantly more opioid analgesics than non-marijuana users, both before (Fig. 1a) and after adjustment (Table 2). Compared to non-marijuana users, opioid use was greater in chronic marijuana users (7.1 vs. 5.7 mg, *p* = 0.049) and episodic users (7.8 mg vs. 5.7 mg, *p* <  0.001). Chronic marijuana users reported similar opioid analgesic consumption compared to episodic marijuana users (*p* = 0.41).

**Table 2:** Mean (standard error) daily opioid consumption and daily pain numeric rating scale (NRS) scores, by marijuana status and other drug use

Outcome, stratified	Marijuana user (*n* = 54)	No marijuana use (*n* = 207)	*p* value
No other drug use^a^	(*n* = 40)	(*n* = 198)	
Mean opioid consumption	8.53 (0.33)	5.86 (0.18)	*< 0.001*
LSM^b^ opioid consumption	7.57 (0.36)	5.65 (0.18)	*< 0.001*
Mean pain NRS score	5.17 (0.15)	4.17 (0.07)	*< 0.001*
LSM^b^ mean pain NRS score	4.92 (0.16)	4.19 (0.11)	*< 0.001*
Other drug use^a^	(*n* = 14)	(*n* = 9)	
Mean opioid consumption	8.81 (0.82)	10.58 (0.97)	0.29
LSM^b^ opioid consumption	5.59 (0.81)	6.10 (1.17)	0.71
Mean pain NRS score	5.02 (0.24)	5.54 (0.25)	0.15
LSM^b^ mean pain NRS score	5.28 (0.34)	6.00 (0.48)	0.07

Analyzed with a repeated measures linear mixed model

*LSM* least square mean

^a^Drug use: amphetamines, barbiturates, benzodiazepines, cocaine, methamphetamine, and opiates

^b^Adjusted for ISS, age, and cause of motor vehicle crash injury

*P* < 0.05 in italic is clinically significant

Pain scores were significantly greater over the hospitalization for marijuana users compared to non-users, both before (Fig. 2a) and after adjustment (Table 2). When the frequency of marijuana use was examined, episodic marijuana users reported higher pain scores than chronic marijuana users (5.3 vs. 4.2, p <  0.001) as well as non-users (5.3 vs. 4.2, *p* <  0.001).

### Results, drug users

Nine percent of patients tested positive for drugs (i.e. amphetamines, barbiturates, benzodiazepines, cocaine, methamphetamine, or opiates). Opioid consumption was not significantly different in drug users who also used marijuana compared to drug users who did not use marijuana, both before (Fig. 1b) and after adjustment (Table 2). However, drug users who chronically used marijuana reported the highest opioid analgesic consumption relative to episodic marijuana use (11.3 mg vs. 4.4 mg, *p* = 0.01) and non-marijuana use (11.3 mg vs. 6.2 mg, *p* = 0.05), after adjustment.

Pain scores were not significantly different in drug users who also used marijuana compared to those who did not use marijuana, both before (Fig. 2b) and after adjustment (Table 2). However, when further examined by frequency of marijuana use, drug users who did not use marijuana reported higher pain scores relative to drug users who used marijuana chronically (6.2 vs. 4.6, p = 0.01) and episodically (6.2 vs. 5.7, *p* = 0.06).

## Discussion

The primary findings from this pilot study suggest that marijuana use significantly affects acute pain management and results in increased consumption of opioid analgesics and greater self-reported pain following traumatic injury, especially in patients who did not report using other drugs. We also identified a low prevalence of other drug use but a relatively high prevalence of chronic marijuana use among trauma patients, especially in trauma centers in Colorado where marijuana has been legalized for both medical and recreational use.

To our knowledge, this is the first study to examine the effect of marijuana use and abuse on acute pain management following traumatic injury. We observed that marijuana’s effect on pain was modified by concomitant drug use. In drug users the addition of marijuana did not appear to effect opioid consumption unless it was used chronically. In non-drug users (representing 91% of our population), opioid administration over the course of the hospital stay was greatest for trauma patients who had used marijuana, both for chronic and episodic use compared to non-marijuana users, even after adjustment for injury severity, age, and specific type of MVC. This translates to a 25–37% increase in opioid consumption for marijuana users than non-marijuana users. Additionally, pain scores were significantly higher in marijuana users compared to non-users, even after adjustment for relevant confounders. The difference in pain scores in marijauna users vs. non-users (5.3 vs. 4.2) is striking when considering that pain scores ≤4 on a 0–10 scale are mild/moderate and scores of ≥5 are considered severe [26].

Prior studies report changes in acute pain management in opioid-tolerant patients, including an increase in opioid consumption [27–29]. Patanwala et al. were the first to prospectively compare the effect of opioid tolerance on the post-surgical analgesic response to opioids, demonstrating a significant increase in opioid consumption and greater pain NRS scores immediately after total knee arthroplasty in opioid tolerant patients relative to the naïve group [28]. Other studies examining opioid tolerance and post-operative pain management reported greater use of analgesics [29], and greater post-surgical requirement for epidural anesthesia [27] in patients who had prior opioid treatment. Neighbor and colleagues examined illegal substance abuse in the emergency department, which included cocaine, heroin, and amphetamine, reporting that substance abusers had significantly higher pain NRS scores compared to non-substance abusers at triage (8.96 vs. 7.81, *p* = 0.003) [30]. We also identified an association between marijuana use and abuse with acute pain management, and much like the opioid tolerant populations, identified chronic use of marijuana resulted in the greatest need for increased analgesia following injury.

Trauma patients commonly have substance abuse issues or other positive toxicology findings. There was a low prevalence of other drug use but a relatively high prevalence of chronic marijuana use, especially in trauma centers in Colorado. In our study 25% were acutely intoxicated, 21% used marijuana, and 9% used other drugs. Marijuana use was reported approximately 4 times more frequently in Colorado hospitals compared to the hospital in Texas. While the study is not designed to point to any causality related to the permissive marijuana laws, we were impressed by the prevalence of marijuana usage amongst our trauma populations. There appears to be a profound increase in marijuana use amongst trauma patients in the states with permissive marijuana laws. It is possible that the increased marijuana use leads to more motor vehicle collisions, but it is not possible to draw this conclusion based upon our data. This study should serve as a call to action for more research into the topic of legalization of marijuana.

We believe the increasing prevalence of marijuana use and other substance abuse issues will have clinical implications for acute pain management. Specifically, these data suggest that patients with marijuana use and abuse issues merit special consideration during acute pain management. These data may help set reasonable expectations for patients regarding the severity and duration of pain they experience, and could help clinicians recognize patients that are more likely to experience suboptimal pain management.

Despite its generally illicit status globally and its schedule I status in the US, there is a growing body of research examining endogenous cannabinoids (endocannabinoids) and exogenous cannabinoids as a target of pharmacotherapy [15]. Several endocannabinoids function to suppress pain sensitivity through their binding to the G-coupled CB1 and CB2 receptors [31]. The activation of these cannabinoid receptors inhibits calcium channels, resulting in activation of potassium channels and decreases in neurotransmitter release from several tissues [32, 33], including inhibition of norepinephrine release from sympathetic nerve terminals and diminished sympathetically mediated pain [15]. This activation of the cannabinoid receptors may be a potential mechanism of action for the antinociceptive effects of cannabinoids [34]. However, the antinociceptive effects of cannabinoids may respond to inflammatory, neurogenic, and chronic pain better than acutely evoked pain [35]. Other studies have demonstrated that the binding of endocannabinioids to CB1 receptors unexpectedly resulted in pain sensitization in in-vivo experiments, and may increase the risk of turning acute pain into chronic pain [36]. Thus, it is plausible that cannabis may be beneficial in treating chronic pain but may be detrimental in the acute pain setting.

There are study limitations. Primarily, this is a pilot study. Some subgroup sizes are low and possibly too small to draw valid conclusions. However, this is a pilot trial and is used as hypothesis generating in order to plan future studies. In the planned prospective study, approximately 360 patients are needed to adequately power the study. Second, the study has many of the disadvantages of a retrospective chart review. For instance, there is the possibility of exposure misclassification: patients were considered non-users if they had no urine drug toxicology screen or a negative toxicology screen and did not self-report using marijuana. Also, marijuana users without details on frequency of use were considered episodic users. Third, our findings might not be generalizable because we excluded minors and we chose to focus on patients who sustained MVC injuries because this population was thought to contain a high concentration of marijuana users. Marijuana consumption also increases the risk of non-traffic injuries, in particular falls in older adults [37], which is a population of interest for future study. Fourth, we excluded patients with a LOS > 14 days (10% of the population), because there is the possibility that the amount of opioids received for pain management over several weeks might lead to acute tolerance and increased opioid consumption unrelated to pre-injury drug and marijuana use [38]. Fifth, 14% of analgesics were non-opioids; we did not report differences in non-opioid analgesics, in part because of there is no standard approach to converting to equianalgesic doses. However, multimodal analgesia can achieve opioid-sparing effects, thereby affecting the data reported for opioid consumption. Lastly, patients who are conscious but non-verbal use a picture face scale, which also utilizes a pain NRS but there are fewer categories: 0 (no pain), 2 (just a little bit) 4 (hurts a little more), 6 (hurts even more), 8 (hurts a whole lot), and 10 (hurts as much as you can imagine). Only the anchor points of 0 and 10 are directly comparable to the pain NRS (0–10). Pain scores may be less accurate in patients with more severe injuries. This limitation should not bias our findings because there were no differences in injury severity (ISS or GCS) between marijuana users and non-users.

## Conclusions

These preliminary data suggest that marijuana use, especially chronic use, may affect pain response to injury by requiring greater frequency and dosing of opioid analgesia. These results were less pronounced in patients who used other drugs. With the increasing prevalence of marijuana use and abuse, and the frequency in which patients with traumatic injury also report substance abuse, our findings have important and potentially well reaching clinical implications. We are planning a larger prospective study to further investigate the relationship between substance use, opioid analgesics, and acute pain management in traumatic injury.

## Abbreviations

ASSIST: Alcohol Smoking and Substance Involvement Screening Test
BAC: Blood alcohol concentration
GCS: Glasgow coma scale (GCS)
ICU: Intensive care unit
ISS: Injury severity score
LOS: Length of stay
MVC: Motor vehicle crash
NRS: Numeric rating score
SBIRT: Screening, brief intervention, and referral to treatment
THC: Tetrahydrocannabinol
UDS: Urine drug screen
